# Calcitriol restrains microglial M1 polarization and alleviates dopaminergic degeneration in hemiparkinsonian mice by boosting regulatory T‐cell expansion

**DOI:** 10.1002/brb3.3373

**Published:** 2024-02-12

**Authors:** Yangzhi Xie, Liang Chen, Jiacheng Chen, Yongjun Chen

**Affiliations:** ^1^ Department of Neurology, The Affiliated Nanhua Hospital, Hengyang Medical School University of South China Hengyang China; ^2^ Department of Intensive Care Unit, The Affiliated Nanhua Hospital, Hengyang Medical School University of South China Hengyang China

**Keywords:** M1/M2 polarization, neuroinflammation, Parkinson's disease, regulatory T cell, vitamin D receptor

## Abstract

**Objective:**

Vitamin D deficiency is a risk factor for Parkinson's disease (PD) and vitamin D supplementation robustly alleviates neurodegeneration in PD models. However, the mechanisms underlying this effect require further clarification. Current evidence suggests that harnessing regulatory T cells (Treg) may mitigate neuronal degeneration. In this study, we investigated the therapeutic effects of vitamin D receptor activation by calcitriol on PD, specifically focusing on its role in Treg.

**Methods:**

Hemiparkinsonian mice model was established through the injection of 6‐OHDA into the striatum. Mice were pretreated with calcitriol before 6‐OHDA injection. The motor performance, dopaminergic neuronal survival, contents of dopamine, and dopamine metabolites were evaluated. The pro‐inflammatory cytokines levels, T‐cell infiltration, mRNA expression of indicated microglial M1/M2 phenotypic markers, and microglial marker in the midbrain were detected. Populations of Treg in the splenic tissues were assessed using a flow cytometry assay. PC61 monoclonal antibody was applied to deplete Treg in vivo.

**Results:**

We show that calcitriol supplementation notably improved motor performance and reduced dopaminergic degeneration in the 6‐OHDA‐induced PD model. Mechanistically, calcitriol promoted anti‐inflammatory/neuroprotective Treg and inhibited pro‐inflammatory/neurodestructive effector T‐cell generation in this model. This process significantly inhibited T‐cell infiltration in the midbrain, restrained microglial activation, microglial M1 polarization, and decreased pro‐inflammatory cytokines release. This more favorable inflammatory microenvironment rescued dopaminergic degeneration. To further verify that the anti‐inflammatory effects of calcitriol are associated with Treg expansion, we applied an antibody‐mediated Treg depletion assay. As predicted, the anti‐inflammatory effects of calcitriol in the PD model were diminished following Treg depletion.

**Conclusion:**

These findings suggest that calcitriol's anti‐inflammatory and neuroprotective effects in PD are associated with its potential to boost Treg expansion.

## Graphical abstract

We explored the therapeutic effects of vitamin receptor activation by calcitriol in a mouse model of PD. We found that calcitriol is potent to protect against neuroinflammation and neuronal degeneration by boosting Treg. Vitamin D receptor may be a therapeutic option for PD.

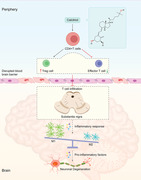

## 1 INTRODUCTION

Parkinson's disease (PD) is a movement disorder clinically characterized by bradykinesia, rigidity, and tremor. The neuropathologic hallmark of PD is the progressive loss of dopaminergic neurons. Multiple mechanisms may be involved in the etiology of PD, including epigenetics (Naren et al., 2023; Tryphena et al., 2023; Uppala et al., 2023), oxidative stress, and neuroinflammation (Rajan et al., 2023). Vitamin D is a fat‐soluble secosteroid that principally functions in promoting calcium absorption. Emerging evidence suggests that the function of vitamin D is not restricted to bone health as the neuroprotective effects of vitamin D have been proposed to limit neurodegenerative diseases, including PD (Pignolo et al., 2022). Past studies have provided clues that vitamin D and vitamin D receptor (VDR) are associated with PD. Vitamin D insufficiency is more prevalent in patients suffering from PD, and the levels of plasma vitamin D are negatively associated with PD severity, suggesting vitamin D insufficiency may be a risk factor for PD (Fullard & Duda, 2020; Wang et al., 2015). In addition, VDR gene polymorphisms were associated with an increased risk of PD (Lv et al., 2020), and germline ablation of VDR resulted in the impairment of motor function in mice (Girgis et al., 2015). In vivo and in vitro evidence also indicates that vitamin D supplementation, or VDR activation, may alleviate dopaminergic neuronal injury (Araújo de Lima et al., 2022; Hu et al., 2021). However, the mechanisms that underpin these effects are poorly understood.

Peripheral T cells have a profound impact on PD. Postmortem and in vivo studies both revealed that T cells are present in the PD brain (Galiano‐Landeira et al., 2020; Williams et al., 2021). Furthermore, Rag1^−/−^ mice, an animal model lacking T cells, were not sensitive to toxin‐induced dopaminergic neuronal loss (Brochard et al., 2009). CD4+ T cells can be divided into two subtypes, specifically, anti‐inflammatory Treg and pro‐inflammatory effector T cells (Teff) (Sakaguchi et al., 2020). Treg/Teff imbalance, which favors inflammation, may facilitate T‐cell infiltration within the neurodegenerative brain (DeMaio et al., 2022). T‐cell infiltration may disturb the immune homeostasis of microglia, thus promoting microglia‐mediated neuroinflammation and neuronal degeneration (MacMahon Copas et al., 2021). Moreover, immunomodulatory agents such as vasoactive intestinal peptide, bee venoms, and granulocyte‐macrophage colony stimulating factor have each shown measurable neuroprotective effects in PD models due to their potential to boost Treg generation (Chung et al., 2012; Mosley et al., 2019; Olson et al., 2020).

Abundant evidence implicates vitamin D involvement in autoimmune diseases such as multiple sclerosis (MS). People in high‐latitude regions are at increased risk to develop MS, and prior studies speculated that light deprivation and vitamin D deficiency may play a role in this effect (Feige et al., 2020). Clinical data also support that vitamin D serves as immunomodulatory agents (Esteves et al., 2022). VDR is widely expressed in immune cells (Handel et al., 2013), and Treg dysfunction has been extensively studied in MS (Verreycken et al., 2022). By targeting the VDR on CD4+ T cells, calcitriol exerted neuroprotective effects in an experimental model of MS by boosting Treg expansion (Galoppin et al., 2022). These studies suggest that VDR activation may be a feasible strategy for Treg expansion; however, no studies have focused on the immunomodulatory role of calcitriol in PD pathophysiology.

In this study, we sought to investigate the therapeutic effects of calcitriol in PD. We hypothesized that the neuroprotective and anti‐inflammatory effects of calcitriol in PD may be associated with its potential to boost Treg expansion.

## 2 MATERIALS AND METHODS

### 2.1 Grouping and treatment

**FIGURE 1:**
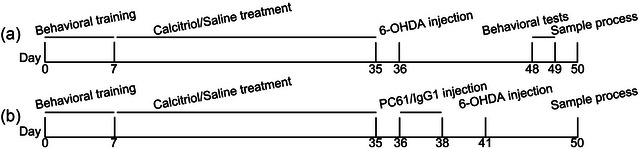
Experimental timeline used in this study.(a) Timeline used for evaluating the therapeutical effects of calcitriol in this indicated PD model. (b) Timeline used for evaluating the therapeutical effects of calcitriol upon Treg depletion in this indicated PD model.

All animal procedures were approved by the Ethics Committee of Affiliated Nanhua Hospital, University of South China. Six to seven‐week‐old male C57BL/6J mice (18–22 g) were kept with ad libitum access to food and water. Mice were trained to adjust a behavioral instrument for 7 days, and mice with poor motor performance were not recruited. Mice were then randomly assigned to either calcitriol (Cal, 3 μg/kg/day, i.p.) treated or vehicle saline‐treated group. After treatment with calcitriol or vehicle for consecutive 29 days, mice were prepared for 6‐OHDA lesioning on day 36 as described previously (Yu et al., 2021). Mice were anesthetized and then placed on a stereotactic system for 6‐OHDA injection. The stereotaxic coordinates for striatum injections were dorsoventral, −3.0; mediolateral, −2.0 mm; anteroposterior, and −0.6 mm from bregma, and a total volume of 3 μL of 6‐OHDA solution (2 μg/μL diluted in PBS with 0.02% sodium l‐ascorbate) was injected into the left striatum. As a control, the sham group was intrastriatal injected with saline. On days 48–49, mice were subjected to behavioral tests. In this section of the experiment (Figure 1a), mice were randomly assigned to Sham, Sham + Cal, 6‐OHDA, and 6‐OHDA + Cal groups. To further verify the neuroprotective effects of calcitriol are associated with Treg expansion, mice were intraperitoneally injected with PC61 anti‐CD25 monoclonal antibody (1 mg/kg/d; Biolegend, catalog: 102002) for consecutive 3 days to deplete Treg in vivo the day after the last calcitriol/saline treatment, and IgG1 was injected as an isotype control. Mice were then prepared for 6‐OHDA lesioning on day 41. In this section of the experiment (Figure 1b), mice were randomly assigned to Sham, 6‐OHDA, and 6‐OHDA + Cal groups. Mice were sacrificed after being anesthetized, and indicated samples were collected for further analysis.

### 2.2 Behavioral tests

A rotarod test was used to evaluate animal coordination and strength. Each animal was placed on an accelerating rotarod (30 rpm), and the latency to fall off the rod was recorded. If the subject did not fall off the rod within 5 min, a maximum value of 300 s was recorded.

A pole test was used to measure animal coordination and grip. Each animal was placed vertically on the top of a rough‐surfaced pole (0.8 cm diameter, 60 cm length) in a head‐up orientation. The elapsed time for each animal to completely turn its head downward was recorded as T‐turn time. The elapsed time for each animal to climb down from the top of the pole to the bottom was recorded as T–D time. If the animal stayed on the pole for over 2 min, it was recorded as the maximum value of 120 s. Each experiment was repeated in triplicate, and the mean was calculated.

### 2.3 Immunohistochemistry and immunofluorescence microscopy

Brains were dissected and post‐fixed in 4% PFA overnight. Samples were then transferred to a 30% sucrose solution until fully equilibrated. Midbrain tissues were coronally sectioned (35 mm). For immunohistochemical staining, sections were incubated with anti‐tyrosine‐hydroxylase (TH) (1:400; Abcam, catalog: ab75875) primary antibody for 48 h. The sections were then incubated with HRP‐conjugated anti‐rabbit IgG (1:50; Beyotime) secondary antibody. For immunofluorescence staining, tissue sections were permeabilized using 0.5% Triton X‐100 in PBS. The sections were incubated with anti‐CD3 antibody (1:100; Proteintech, catalog: 17617‐1‐AP) overnight. Sections were subsequently incubated with CY3‐labeled anti‐mouse secondary antibody (1:800; Abcam). Stained tissues were observed using a confocal fluorescence microscope (Leica).

### 2.4 Liquid chromatography tandem mass spectrometry

Liquid chromatography tandem mass spectrometry (LC–MS/MS) was used to evaluate the content of dopamine (DA) as well as the dopamine metabolites dihydroxy‐phenyl acetic acid (DOPAC) and homovanillic acid (HVA) in the striatum. Tissue samples were ground in 500 μL of 80% methanol for 5 min and subsequently sonicated for 30 min at 4°C and let stand for an additional hour. After centrifugation at 12,000 rpm for 15 min, the supernatant was collected and filtered through a 0.22 μm mesh prior to preparation for LC–MS/MS analysis. The various apparatuses used for LC–MS/MS analysis were as follows: liquid chromatography (Water Acuity UPLC); mass spectrography (AB SCIEX 5500 Qtrap‐MS); and chromatographic column (Acquity UPLC HSS T3; 1.8 μm, 2.1 mm × 100 mm).

### 2.5 Quantitative real‐time PCR

TRIzol reagent (Sigma‐Aldrich) was used to extract total RNA from the midbrain. Afterward, RNA was reverse transcribed to cDNA using a PrimeScript RT reagent kit (Takara). Real‐time PCR was conducted using the SYBR Premix EX Taq I (Takara) on a 7300 Plus Real‐time PCR System (Thermo Fisher). Each reaction mixture contained 1 μL of cDNA template, 5 pmol primer, 10 μL PCR master mixture, and water. The thermocycling conditions used were an initial 95°C for 15 s, followed by 40 cycles at 94°C for 10 s and 60°C for 25 s. The relative mRNA levels of genes analyzed were obtained using GAPDH mRNA as the internal standard. The comparative threshold cycle method was used to analyze the data. The primer sequences are shown in Table 1.

**TABLE 1:** Primer sequence of targeted genes.

Target gene	Forward primer sequence	Reverse primer sequence
GAPDH	GCCAAGGCTGTGGGCAAGGT	TCTCCAGGCGGCACGCAGA
CD32	AATCCTGCCGTTCCTACTGATC	GTGTCACCGTGTCTTCCTTGAG
iNOS	GGTGAAGGGACTGAGCTGTT	ACGTTCGTTCTCTTGCA
TNF‐α	CAGGCGGTGCCTATGTCTC	CCATTTGGGAACTTCTCATCCCTT
CD206	AAGGAAGGTTGGCATTTGT	CTTTCAGTCCTTTGCAAGC
Arg‐1	CACCTGAGCTTTGATGTCG	TGAAAGGAGCCCTGTCTTG
IL‐10	CCAAGCCTTATCGGAAATGA	TTTTCACAGGGGAGAAATCG
IBA‐1	GCAATTCCTCGATGATCCCAAA	GATCAAACTCCATGTACTTCACCTT

### 2.6 Enzyme‐linked immunosorbent assay

Commercially available ELISA kits (Beyotime) were used to measure pro‐inflammatory cytokines such as TNF‐α, IL‐6, and IL1‐β in the mesencephalic tissues. All procedures were strictly conducted following manufacturer's instructions.

### 2.7 Flow cytometric analysis

Splenic tissues were ground and passed through a filter mesh. Cell suspensions were incubated with an equal volume of lymphocyte separation liquid (Solarbio, catalog: P8620) to harvest splenocytes per the manufacturer's instructions. Splenocytes were subsequently resuspended in a buffer solution. For analysis of the Treg subset, splenocytes were labeled with antibodies against CD4 (eBioscience, catalog: 11‐0042‐82), CD25 (eBioscience, catalog: 15‐0251‐82), and FOXP3 (eBioscience, catalog: 17‐5773‐82). The flow cytometer (CytoFLEX, Beckman) was gated on CD4+ T cells for analysis of Treg.

### 2.8 Statistics

All data were analyzed by SPSS 22.0 and were expressed as mean ± SEM. Data were analyzed using one‐way ANOVA with Tukey's posttest. *p* < .05 were considered statistically significant.

## 3 RESULTS

### 3.1 Calcitriol pretreatment improves the motor performance of hemiparkinsonian mice

**FIGURE 2:**
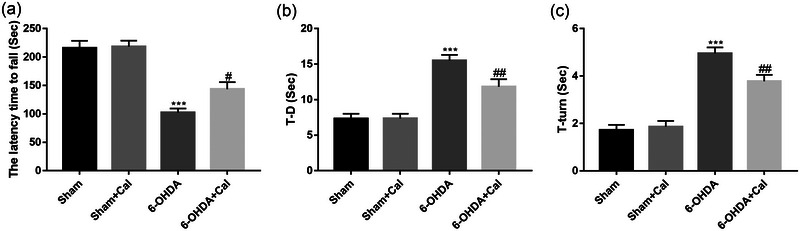
Calcitriol induced functional improvement in hemiparkinsonian mice. (a) The latency time prior to falling off a rotating rod was recorded in the rotarod test. (b) In the pole test, the time for each animal to climb from the top to the bottom was recorded as T–D. (c) In the pole test, the time for each animal to turn its head completely downward was recorded as T‐turn. *n* = 10 per group. ***p < .001 compared to the sham group. ^#^ *p* < .05, ^##^ *p* < .01, compared to the 6‐OHDA injection group. Data were analyzed by one‐way ANOVA with Tukey's posttest.

To evaluate whether calcitriol treatment induces a functional improvement in the hemiparkinsonian mouse model, mice were subjected to the rotarod and pole test. ANOVA revealed that the motor performance significantly differed among the groups (rotarod test: *F*(3, 36) = 26.9, *p* < .001; pole test: *F*(3, 36) = 23.1, *p* < .001). In the rotarod test, mice injected with 6‐OHDA exhibited a lower latency to fall off the rotating rod compared to mice in the sham group (*p* < .001, Figure 2a). Of note, mice pretreated with calcitriol prior to 6‐OHDA injection exhibited a higher latency to fall than the 6‐OHDA only group (*p* = .012, Figure 2a).

In the pole test, mice injected with 6‐OHDA exhibited poorer performance compared to the sham group (TD: *p* < .001; T‐turn: *p* < .001; Figure 2b,c). While compared to the 6‐OHDA only group, mice pretreated with calcitriol prior to 6‐OHDA injection exhibited improved performance in the pole test (TD: *p* = .003; T‐turn: *p* = .002; Figure 2b,c). These data suggest that calcitriol treatment induced a functional improvement in the 6‐OHDA‐induced PD model.

### 3.2 Calcitriol pretreatment protects dopaminergic neurons in the substantia nigra against 6‐OHDA neurotoxicity

**FIGURE 3:**
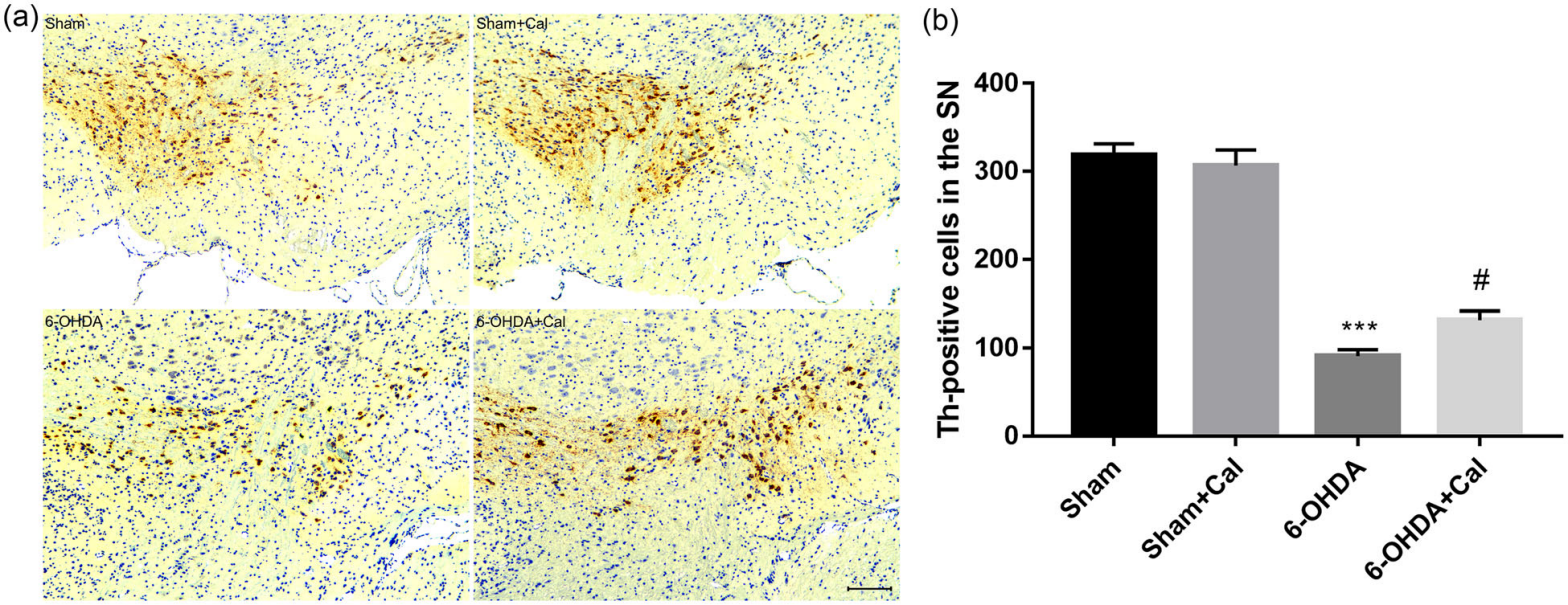
Calcitriol treatment alleviated dopaminergic neuronal injury in the SN. (a) Representative images showing tyrosine‐hydroxylase (TH) immunostaining of dopaminergic neurons within the SN in indicated animal models. Scale bar 100 μm. (b) The number of TH‐positive cells in the SN was counted, *n* = 4 per group. ***p < .001 compared to the sham operation group. ^#^ *p* < .05 compared to the 6‐OHDA injection group. Data were analyzed by one‐way ANOVA with Tukey's posttest.

To evaluate whether calcitriol pretreatment protects against 6‐OHDA‐induced dopaminergic neuronal loss, mesencephalic tissues were collected for TH immunostaining (Figure 3a). According to ANOVA, TH‐positive cells in the substantia nigra were significantly different among the groups (*F*(3, 12) = 12.8, *p* < .001). We observed that 6‐OHDA injection resulted in a reduced number of TH‐positive dopaminergic neurons in the substantia nigra to the sham group (*p* < .001, Figure 3b), whereas pretreatment with calcitriol prior to 6‐OHDA injection protected against this 6‐OHDA‐induced dopaminergic neuronal loss (*p* = .034, Figure 3b).

### 3.3 Calcitriol pretreatment increases the level of dopamine and its metabolites in the striatum of hemiparkinsonian mice

**FIGURE 4:**
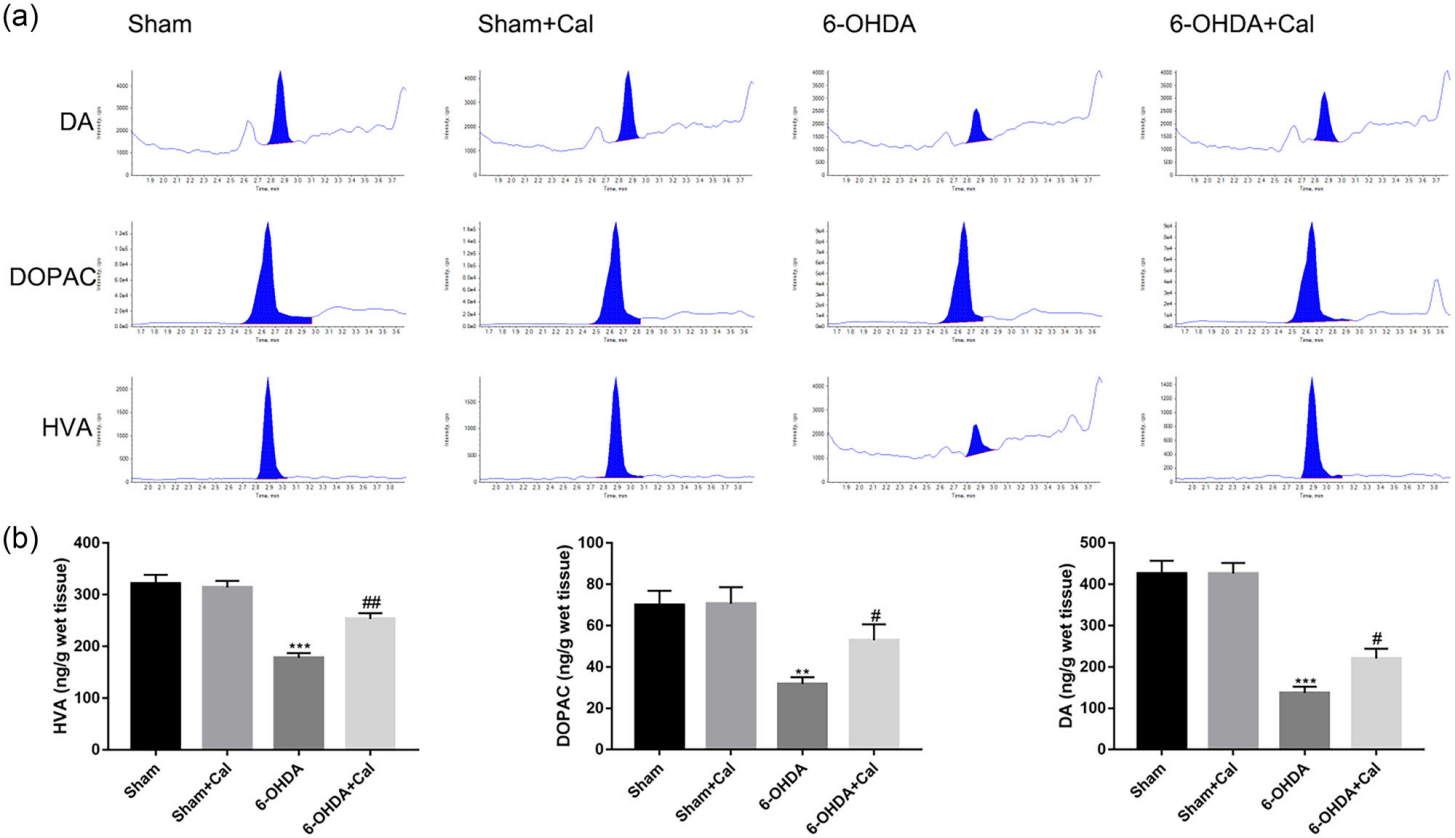
Calcitriol pretreatment increased the content of DA, and metabolites dihydroxy‐phenyl acetic acid (DOPAC) and homovanillic acid (HVA), in the striatum of hemiparkinsonian mice. (a) Quantitative analysis of DA, DOPAC, and HVA in the striatum using liquid chromatography tandem mass spectrometry (LC–MS/MS). (b) DA, DOPAC, and HVA abundance in the striatum. *n* = 4 per group. **p < .01, ***p < .001 compared to the sham operation group. ^#^ *p* < .05, ^##^ *p* < .01, compared to the 6‐OHDA injection group. Data were analyzed by one‐way ANOVA with Tukey's posttest.

We next examined the content of DA and DA metabolites, HVA and DOPAC, in the mouse striatum of indicated models using LC–MS/MS (Figure 4a). According to ANOVA, the abundance of DA and DA metabolites were significantly different among the groups (DA: *F*(3,12) = 35.3, *p* < .001; DOPAC: *F*(3, 12) = 7.4, *p* < .001; HVA: *F*(3,12) = 26.7, *p* < .001). As demonstrated in Figure 4b, when compared to the sham group, 6‐OHDA injection resulted in a decreased abundance of DA (*p* < .001), DOPAC (*p* = .002), and HVA (*p* < .001) in the striatum. However, calcitriol pretreatment prior to 6‐OHDA injection significantly increased the content of DA (*p* = .035), DOPAC (*p* = .047), and HVA (*p* = .0014) in hemiparkinsonian mouse brain.

### 3.4 Calcitriol pretreatment restrains microglial polarization towards a pro‐inflammatory phenotype

**FIGURE 5:**
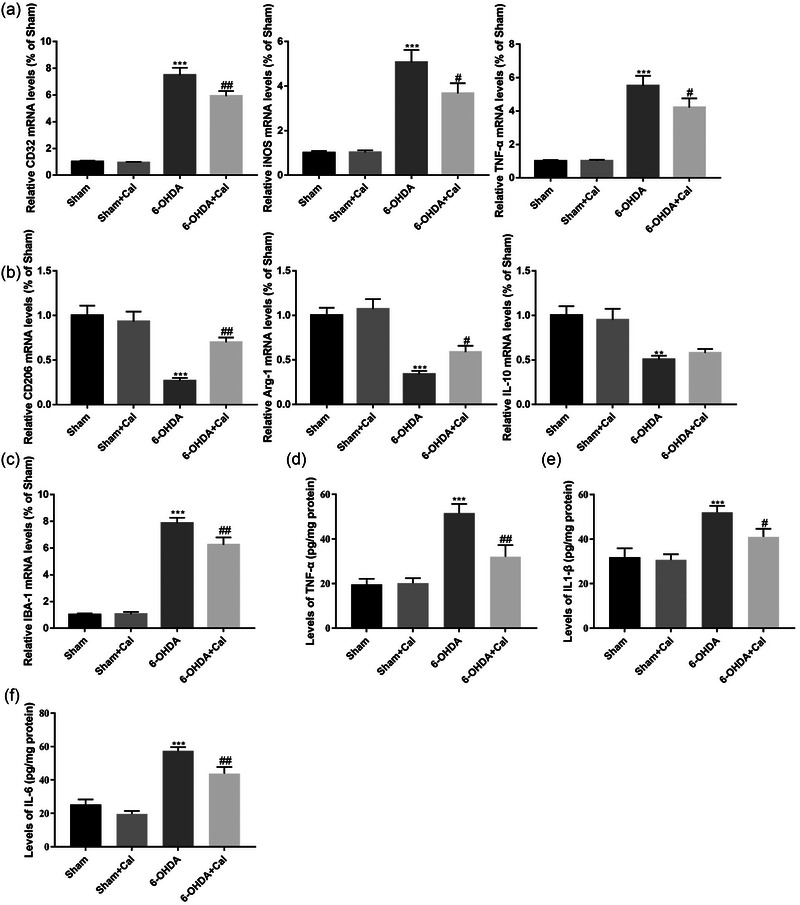
Calcitriol regulated microglial M1/M2 polarization and reduced pro‐inflammatory cytokines release. (a) Relative mRNA expression of indicated microglial M1 phenotypic markers in mesencephalic tissues. (b) Relative mRNA expression of indicated microglial M2 phenotypic markers in mesencephalic tissues. (c) Relative mRNA expression of IBA‐1. (d) Levels of TNF‐α in mesencephalic tissues. (e) Levels of IL1‐β in mesencephalic tissues. (f) Levels of IL‐6 in mesencephalic tissues. n = 5 per group. **p 〈 .01, ***p 〈 .001 compared to the sham operation group. #p 〈 .05, ##p 〈 .01, compared to the 6‐OHDA injection group. Data were analyzed by one‐way ANOVA with Tukey's posttest.

Microglial M1/M2 imbalance favoring the pro‐inflammatory M1 phenotype plays a pivotal role in neuronal degeneration. To evaluate the potential anti‐inflammatory effects of calcitriol in this PD model, we measured the mRNA level of the M1 markers CD32, iNOS, and TNF‐α (Figure 5a), M2 phenotypic markers CD206, Arg‐1, and IL‐10 (Figure 5b), and microglial marker IBA‐1 (Figure 5c) in mesencephalic tissues using real‐time PCR. Pro‐inflammatory cytokines levels in these tissues were measured using ELISA (Figure 5d–f). ANOVA analysis revealed that the levels of M1/M2 markers (CD32: *F*(3, 16) = 98.4, *p* < .001; iNOS: *F*(3, 16) = 31.7, *p* < .001; TNF‐α: *F*(3, 16) = 31.2, *p* < .001; CD206: *F*(3, 16) = 15.3, *p* < .001; Arg‐1: *F*(3, 16) = 18.5, *p* < .001; IL‐10: *F*(3, 16) = 8.3, *p* < .001), IBA‐1 (*F*(3, 16) = 45.1, *p* < .001) and pro‐inflammatory cytokines (TNF‐α: *F*(3, 16) = 16.3, *p* < .001; IL‐1β: *F*(3, 16) = 8.9, *p* < .001; IL‐6: *F*(3, 16) = 35.7, *p* < .001) in mesencephalic tissues were significantly different among the groups. Compared to the sham group, 6‐OHDA injection promoted pro‐inflammatory M1 polarization marker mRNA (CD32: *p* < .001; iNOS: *p* < .001; TNF‐α: *p* < .001; Figure 5a) and inhibited anti‐inflammatory M2 polarization marker mRNA (CD206: *p* < .001; Arg‐1: *p* < .001; IL‐10: *p* = .001; Figure 5b) in the mesencephalic tissues. In contrast, calcitriol administration prior to 6‐OHDA injection restrained M1 polarization mRNA markers (CD32: *p* = .005; iNOS: *p* = .015; TNF‐α: *p* = .039; Figure 5a) and promoted M2 polarization mRNA markers (CD206: *p* = .002; Arg‐1: *p* = .045; IL‐10: *p* = .557; Figure 5b). Calcitriol pretreatment prior to 6‐OHDA injection significantly inhibited microglial activation, as evidenced by lower expression of IBA‐1 (*p* = .004, Figure 5c). Following 6‐OHDA injection, levels of pro‐inflammatory cytokines were significantly increased in the PD model (TNF‐α: *p* < .001; IL‐1β: *p* < .001; IL‐6: *p* < .001; Figure 5d–f), whereas calcitriol pretreatment prior to 6‐OHDA injection significantly reduced pro‐inflammatory cytokines release (TNF‐α: *p* = .002; IL‐1β: *p* = .033; IL‐6: *p* = .004; Figure 5d–f). These data suggest that calcitriol treatment improved the inflammatory microenvironment in mesencephalic tissues dissected from this PD model.

### 3.5 Calcitriol treatment inhibits T‐cell infiltration within the midbrain in the PD model

**FIGURE 6:**
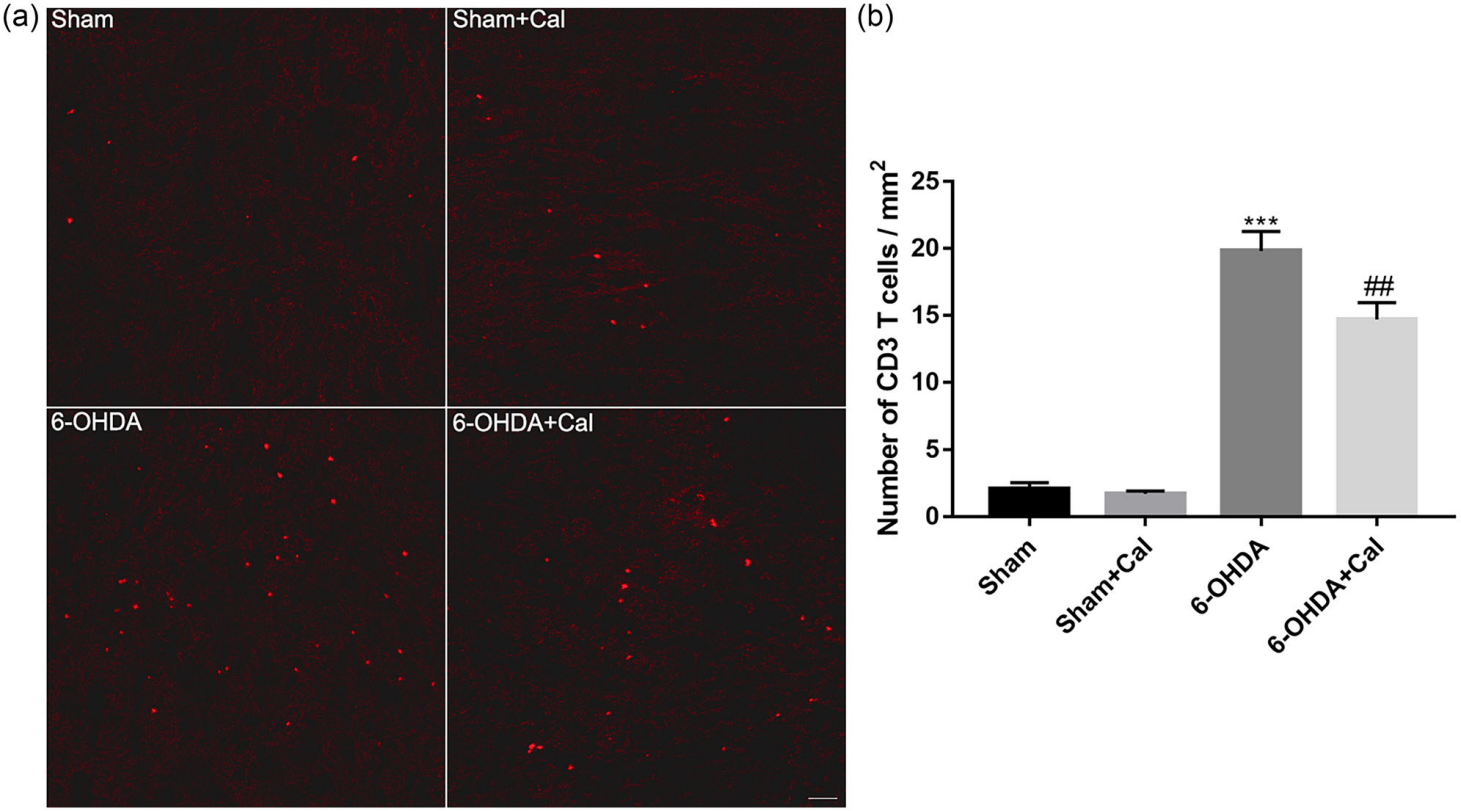
Calcitriol pretreatment restrained T‐cell infiltration in the midbrain of the Parkinson's disease (PD) model. (a) Representative immunofluorescence staining for CD3 T cells in the midbrain. Every eighth serial section was selected for immunofluorescence staining. Scale bar 100 μm. (b) Bar graph showing the number of CD3+ T cells in the midbrain of indicated experimental groups. *n* = 4 per group. ***p < .001 compared to the sham operation group. ##*p* < .01 compared to the 6‐OHDA injection group. Data were analyzed by one‐way ANOVA with Tukey's posttest.

Emerging evidence indicates that infiltrating T cells are associated with neuroinflammation and dopaminergic degeneration. To determine whether calcitriol treatment may limit T‐cell infiltration within the midbrain in this PD model, we analyzed the T‐cell marker CD3 by 9 immunofluorescence staining (Figure 6a) and counted the number of CD3+ cells (Figure 6b). ANOVA revealed that the numbers of CD3+ cells were significantly different among the groups (*F*(3, 12) = 84.9, *p* < .001). Compared to the sham group, mice injected with 6‐OHDA showed increased T‐cell infiltration in the midbrain (*p* < .001, Figure 6b), yet calcitriol pretreatment prior to 6‐OHDA injection significantly inhibited T‐cell infiltration in the midbrain of the PD model (*p* = .003, Figure 6b).

### 3.6 Calcitriol pretreatment boosts splenic Treg expansion

**FIGURE 7:**
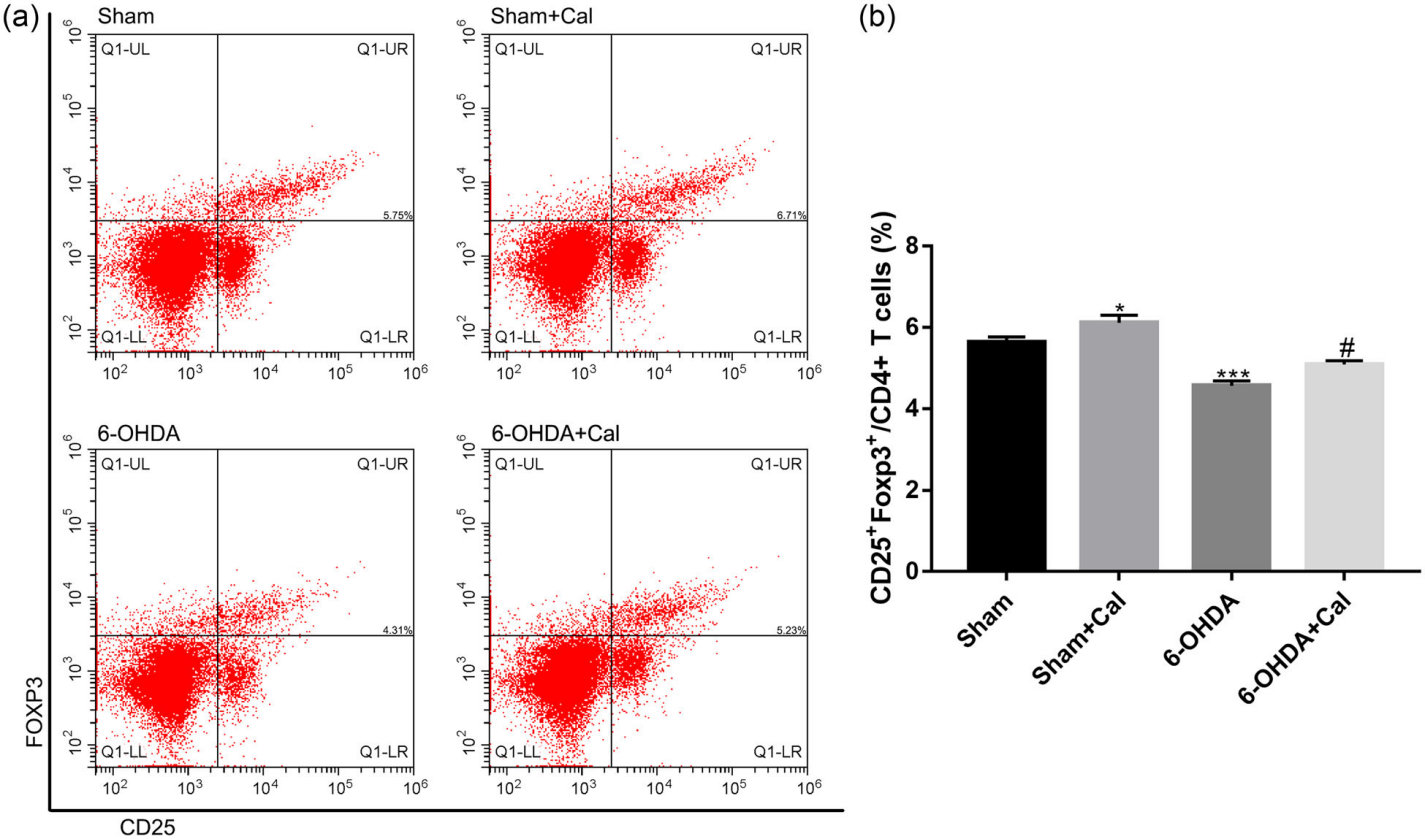
Calcitriol pretreatment promotes splenic Treg generation. (a) The proportion of Treg in CD4+ T cells was measured by flow cytometry in each of the indicated models. (b) Bar graph showing the percentage of Treg/CD4+ T cells measured in splenocyte populations obtained from indicated models. *n* = 5 per group. *p < .05, ***p < .001 compared to the sham operation group. ^#^ *p* < .05 compared to the 6‐OHDA injection group. Data were analyzed by one‐way ANOVA with Tukey's posttest.

An imbalance between Treg and Teff is associated with T‐cell infiltration in PD. To examine whether calcitriol pretreatment upregulates Treg expansion in vivo, we evaluated Treg populations in the splenocytes using flow cytometry (Figure 7a). ANOVA revealed that the frequencies of Treg were significantly different among the groups (*F*(3, 16) = 23.3, *p* < .001). Compared to the sham group, the populations of triple‐positive Treg were found to be relatively lower in the 6‐OHDA treated group (*p* < .001, Figure 7b). In contrast, calcitriol pretreatment prior to 6‐OHDA injection significantly increased Treg populations (*p* = .016, Figure 7b). Calcitriol also increased Treg populations in the sham group (*p* = .027, Figure 7b).

### 3.7 Treg depletion counteracts the anti‐inflammatory effects of calcitriol in hemiparkinsonian mice

**FIGURE 8:**
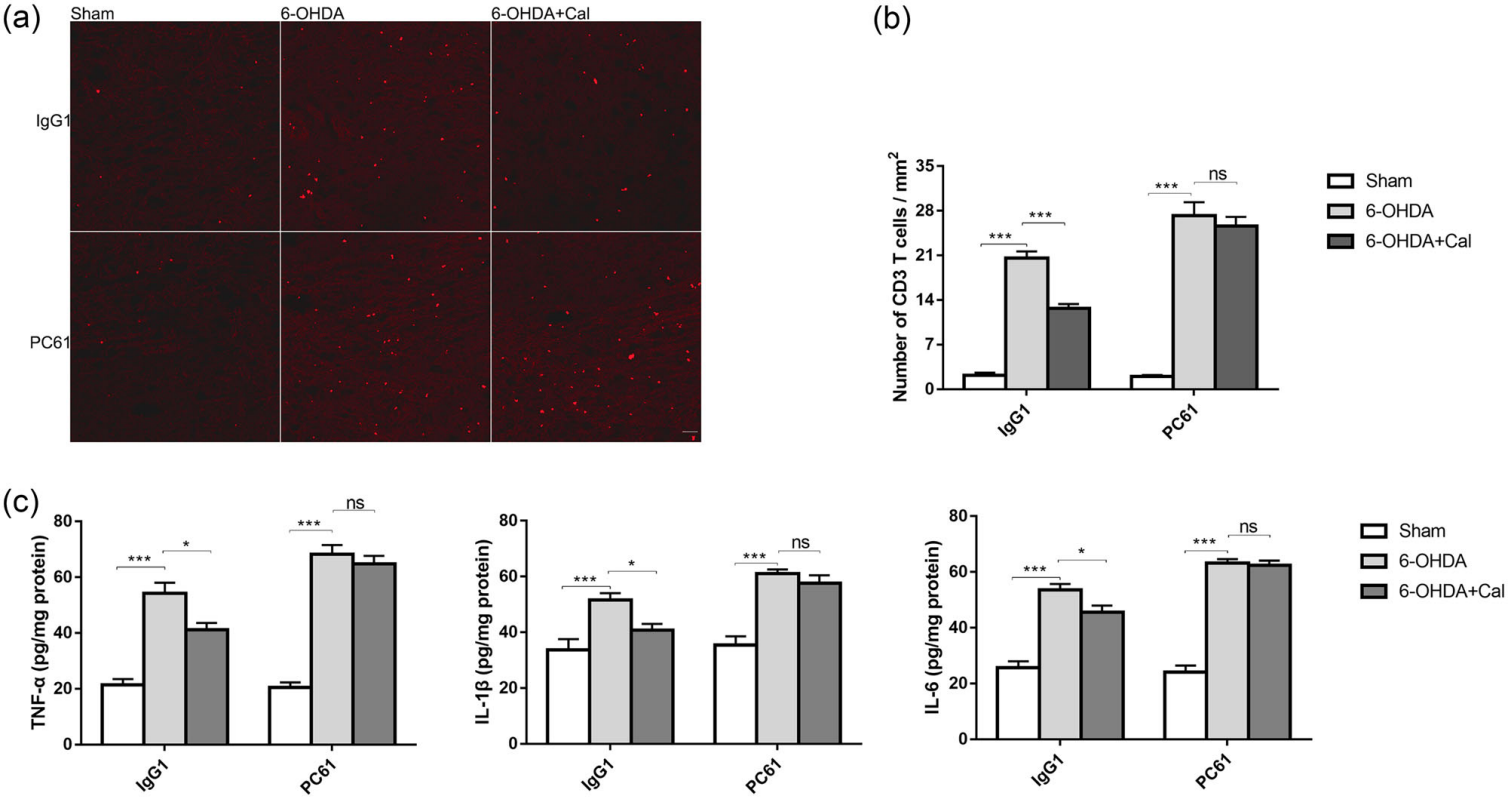
The anti‐inflammatory effects of calcitriol diminished upon Treg depletion. (a) Representative immunofluorescent staining for CD3 T cells in the midbrain. Every eighth serial section was selected for immunofluorescence staining. Scale bar 100 μm. (b) The number of CD3 T cells counted in the midbrain, *n* = 4 per group. (c) Levels of pro‐inflammatory cytokines in the mesencephalic tissues as measured by ELISA, *n* = 5 per group. *p < .05, **p < .01, ***p < .001. Data were analyzed by one‐way ANOVA with Tukey's posttest. ns, not significant.

To validate that the anti‐inflammatory effects of calcitriol are associated with Treg, mice were injected with PC61 anti‐CD25 monoclonal antibody for 3 consecutive days before 6‐OHDA injection prior to analysis by fluorescence microscopy (Figure 8a). Mouse IgG1 was injected as an isotype control. ANOVA revealed that the numbers of CD3+ cells were significantly different among the groups (PC61: (*F*(2, 9) = 149.8, *p* < .001); IgG1: (*F*(2, 9) = 84.7, *p* < .001). As shown (Figure 8b), pretreatment with calcitriol combined with control IgG1 prior to 6‐OHDA injection remarkably restrained T‐cell infiltration within the midbrain (*p* < .001). Following Treg depletion via anti‐CD25 injection, no difference was observed between 6‐OHDA and 6‐OHDA + Cal group (*p* = .60).

We further examined the level of pro‐inflammatory cytokines in mesencephalic tissues by ELISA (Figure 8c). The levels of indicated pro‐inflammatory cytokines TNF‐α (PC61: (*F*(2, 12) = 91.8, *p* < .001); IgG1: *F*(2, 12) = 32.4, *p* < .001), IL‐1β (PC61: (*F*(2, 12) = 28.3, *p* < .001); IgG1: *F*(2, 12) = 9.6, *p* < .001), and IL‐6 (PC61: *F*(2, 12) = 136.9, *p* < .001; IgG1: *F*(2, 12) = 39.8, *p* < .001) were significantly different among the groups. As expected, pretreatment with calcitriol combined with IgG1 before 6‐OHDA injection significantly inhibited pro‐inflammatory cytokine release (TNF‐α: *p* = .011; IL‐1β: *p* = .03; IL‐6: *p* = .03). Following Treg depletion, no significant difference in 6‐OHDA‐induced pro‐inflammatory cytokine release was observed between 6‐OHDA and 6‐OHDA + Cal group (TNF‐α: *p* = .79; IL‐1β: *p* = .66; IL‐6: *p* = .96). These data suggest that the anti‐inflammatory effects of calcitriol were associated with Treg expansion.

## 4 DISCUSSION

**FIGURE 9:**
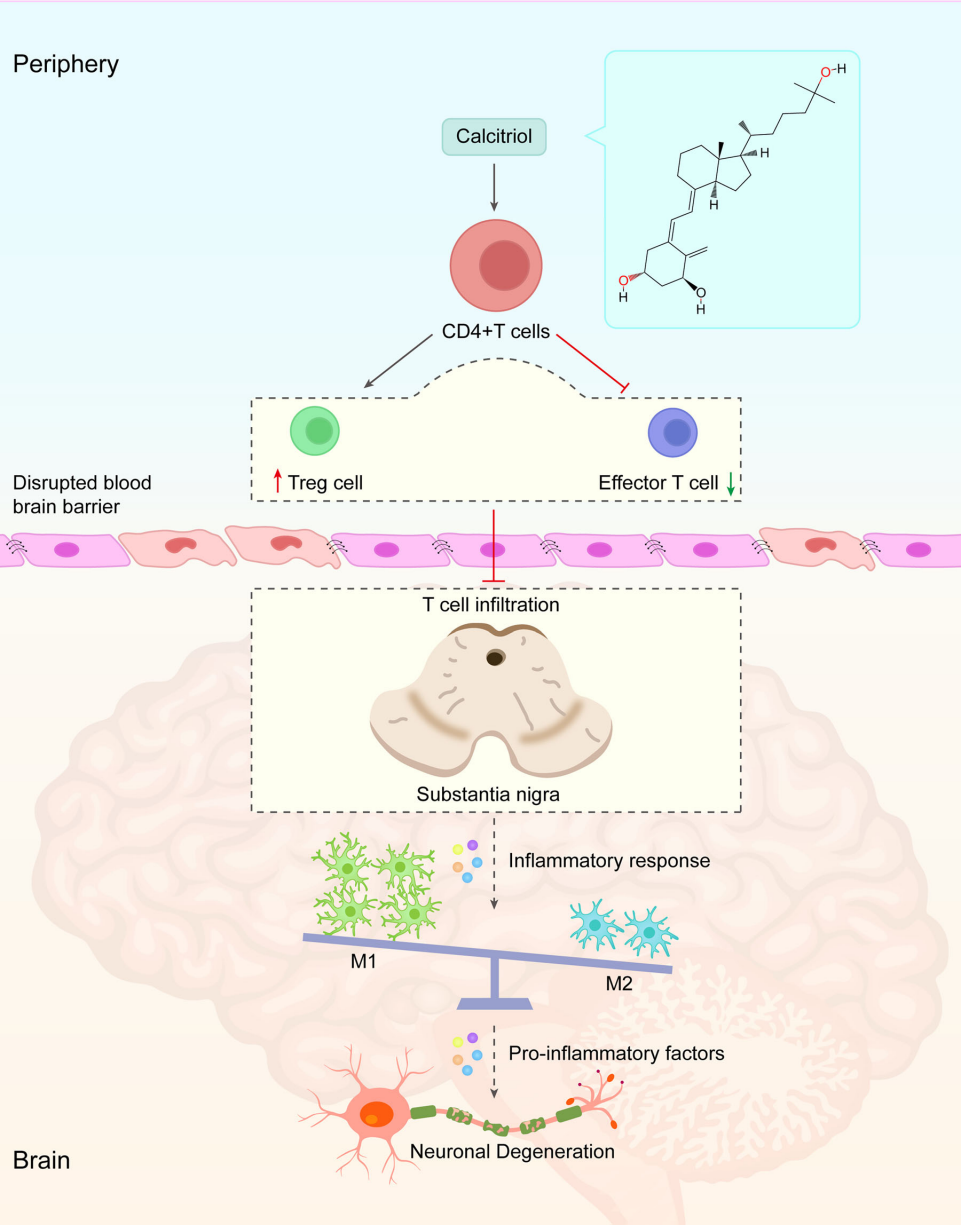
Proposed model outlining the mechanism for calcitriol against neuroinflammation and dopaminergic neuronal degeneration.

In this study, we observed that calcitriol treatment induced functional improvement, protected against dopaminergic neurodegeneration, restrained microglial M1 polarization, and inhibited pro‐inflammatory cytokines release in this PD model. Mechanistically, we found that calcitriol boosted anti‐inflammatory Treg generation. Remarkably, this process restrained T‐cell infiltration thus improving the inflammatory microenvironment. Further, this improvement in the inflammatory microenvironment shifted microglial M1/M2 polarization and promoted dopaminergic neuronal survival (see Figure 9).

An increasing body of evidence suggests that immunological dysfunction in the brain or periphery may function in PD pathobiology, whereas the potential effects of peripheral immune cells such as T lymphocytes remain under exploration. Our results indicate that excessive T‐cell infiltration was found in the PD model used in this study. Preclinical studies indicated that the presence of T cells provides pro‐inflammatory actions within the PD brain (Rauschenberger et al., 2022). Several mechanisms have been described that T cells contribute to the pathophysiology of PD. This immune cell population may directly interact with neurons through LFA1/ICAM binding or Fas/Fas ligand signaling, thus triggering neuronal death (Gérard et al., 2021; Golstein & Griffiths, 2018). Our findings suggest that T‐cell infiltration may indirectly lead to neuronal death by shifting the microglial polarization. In accordance, other studies support the view that infiltrating T cells may be responsible for M1 polarization (Lan et al., 2017). Microglia‐mediated neuroinflammation is a common outcome in PD. Our results suggest that microglia is activated in this indicated rodent model of PD. As a neurotoxic agent for dopaminergic neuron, 6‐OHDA injection induces mitochondrial dysfunction and oxidative stress which are sufficient to activate microglia. T‐cell/microglia crosstalk may produce positive feedback that creates a pro‐inflammatory microenvironment and facilitates neuronal injury. On one hand, activated microglia potently recruited T cells from the periphery to the neurodegenerative brain, and microglia depletion robustly inhibited T‐cell infiltration within the brain (Chen et al., 2023). In contrast, cytokines such as IFN‐γ released from infiltrating T cells may further exacerbate the pro‐inflammatory effects of microglia (Garber et al., 2019). Of note, a recent study indicated that T cells were principally distributed near dopaminergic neurons, suggesting that T‐cell infiltration may be site specific (Karikari et al., 2022). Hence, inhibiting T‐cell infiltration may benefit PD patients.

The prior study implicated that a T‐cell subset imbalance, which favors the pro‐inflammatory Teff, plays a vital role in PD pathology. However, Treg can restrain the function of Teff to maintain immune tolerance (Machhi et al., 2020). In this study, VDR activation by calcitriol promoted Treg generation. In accordance with these findings, prior studies also verified the effects of vitamin D and VDR activation on Treg generation (Moore et al., 2018; Savastio et al., 2020). Calcitriol was reported to boost Treg and inhibit Teff expansion through several mechanisms. VDR is expressed in dendritic cells, the most powerful of the antigen‐presenting cells currently known. Along with the antigen‐presenting process, T cells are differentiated into diverse phenotypes (Hu & Wan, 2011). An in vitro study reported that 1,25(OH)2D3, the active form of vitamin D, enhanced anti‐inflammatory cytokines, such as IL‐10, and restrained pro‐inflammatory cytokines such as TNF‐α and IL‐12 resulting in the release of dendritic cells (Vanherwegen et al., 2019). This process likely triggers the expansion of Treg and apoptosis of Teff, thus inducing immune tolerance. Moreover, the direct immunomodulatory effects of VDR activation on CD4+ T cells have been described in prior studies. VDR expression is significantly elevated in T lymphocytes in an activated state (Fletcher et al., 2022). Calcitriol can inhibit effector Th17 expansion and IL‐17 release by suppressing transcription of target genes such as NFAT, RUNx1, and RORγt (Joshi et al., 2011; Palmer et al., 2011). Similarly, the number and functional alterations of Treg following calcitriol treatment also appear to be regulated at the transcriptional level. Calcitriol is reported to enhance FOXP3 expression via the upregulation of Helios, a positive regulator of FOXP3 (Nashold et al., 2013). Other types of CD4+ T cells, such as Th1 and Th2, are also modulated by VDR activity (Chauss et al., 2022; Song et al., 2018). Of note, peripheral Treg population get affected by intrastriatal 6‐OHDA injection, this might be explained by the fact that misfolded protein or self‐antigen produced by injuring neuronal cells are drained to the peripheral lymphoid nodes by meningeal lymphatic vessels, and they are recognized by antigen presenting cells. Upon the recognition of cognate antigen, naïve T cells are differentiated into antigen‐specific Teff, thus breaking Treg/Teff balance.

Clinical evidence indicates that low serum vitamin D concentrations are associated with PD prevalence; however, the therapeutic effects of vitamin D and its analog on PD patients remain controversial. This may partially be explained by the fact that geographic and/or ethnic differences may yield distinctly different results. Large multicenter randomized trials are required to verify the preventive effects of vitamin D and its analogs to treat PD. Limitations still exist in this study, in vitro studies using purified CD4+ T cells and VDR knockout CD4+ T cells may better illustrate the immunomodulatory effects of calcitriol.

## 5 CONCLUSION

Our findings indicate that VDR activation by calcitriol potently protects against microglial‐mediated neuroinflammation and neuronal degeneration in 6‐OHDA‐induced hemiparkinsonian mice, and that this observed effect appears to be mediated by boosting Treg expansion.

## AUTHOR CONTRIBUTIONS

*Concept and design; drafting of the article*: Liang Chen and Yangzhi Xie. *Data collection and analysis*: Jiacheng Chen. *Critical revision of the article for important intellectual content*: Liang Chen. *Study supervision*: Yongjun Chen. All the authors approved the final article.

## CONFLICT OF INTEREST STATEMENT

The authors declare no conflicts of interest.

### PEER REVIEW

The peer review history for this article is available at https://publons.com/publon/10.1002/brb3.3373.

## Data Availability

Data available upon reasonable request.
